# Supplementary material for: NHR-49/PPAR-α and HLH-30/TFEB cooperate for C. elegans host defense via a flavin-containing monooxygenase
Source: eLife. 2021 May 12;10:e62775. doi: 10.7554/eLife.62775 (PMC8139828; doi:10.7554/eLife.62775)
Supplement: Supplementary file 5. [file elife-62775-supp5.docx]

**List of oligos, crRNAs, and repair templates used in this work.**

| **Oligonucleotides** | **Source** |
| --- | --- |
| *fmo-2(FAD)* crRNA1=  5’ AACAAGCGTGTTGCTGTCAT 3’  *fmo-2(FAD)* crRNA2=  5’ GTCATAGGAGCTGGTGCTTC 3’ | IDT |
| *fmo-2(FAD)* repair template=  5’ cgtgtttgttgtcaaaATGGGGAACAAGCG  TGTTGCTGTCATcGcAGCTGcTGCTTCcGcATTACCGTCGATTCGgtttgtaattctgatttttattgaaataatag 3’ | IDT |
| *fmo-2(NADPH)* crRNA1=  5’ TCACAAGGGTTATGAAGACA 3’  *fmo-2(NADPH)* crRNA2=  5’ TCACGATTACAAGGATCACA 3’ | IDT |
| *fmo-2(NADPH)* repair template=  5’ CAAAGGACGTATTGTTCATTCTCAC  GATTACAAGGAcCAtAAaGGTTATGAAGAtAAaGTAGTTGTTGTCGTTGcAATTGcAAATAGTGGAATCGACGTGGCAGTTGAGCAATCAAGAATTGC 3’ | IDT |
| *snb-1* RT-PCR/F=  5’ GAATCATGAAGGTGAACGTGG 3’  *snb-1* RT-PCR/R=  5’ GAATGACGACGATAGCGCAC 3’ | IDT |
| *fmo-2* RT-PCR/F=  5’ ATAATGAACACGCGTTTCTTC 3’  *fmo-2* RT-PCR/R=  5’ GATGTTTGGCTTGATTCTGA 3’ | IDT |
| *hlh-30* RT-PCR/F=  5’ GAACACATCAGAAGACATGAAAC 3’  *hlh-30* RT-PCR/R=  5’ AAGATGCGATGGCGGGACCT 3’ | IDT |
| *nhr-49* RT-PCR/F=  5’ TCCGAGTTCATTCTCGACG 3’  *nhr-49* RT-PCR/R=  5’ GGATGAATTGCCAATGGAGC 3’ | IDT |
| *lys-5* RT-PCR/F=  5’ GCCAGAGCTGCTGGCCTCAC 3’  *lys-5* RT-PCR/R=  5’ GCCTTTGCTTCACTGACCATTGC 3’ | IDT |
| *clec-60* RT-PCR/F=  5’ CTTTGCTGCAAGTGAACTGTTTC 3’  *clec-60* RT-PCR/R=  5’ GGACATAATCGTGTTTGTTCG 3’ | IDT |
| *H02F09.3* RT-PCR/F=  5’ CGACAAACACCCCTGATAGC 3’  *H02F09.3* RT-PCR/R=  5’ GTGGTTGTGTGGATGATGAC 3’ | IDT |
| *ech-9* RT-PCR/F=  5’ GAAAGAAAATGACACTGAAATG 3’  *ech-9* RT-PCR/R=  5’ ACCGAGAATAAACATGATATC 3’ | IDT |
| *Y65B4BR.1* RT-PCR/F=  5’ ATCTTTACATGGATGCTCAGCAG 3’  *Y65B4BR.1* RT-PCR/R=  5’ GGCCTAGTTTTGAGAAATGGAAG 3’ | IDT |
| *C50F7.5* RT-PCR/F=  5’ CATCCGAAGATCCTCAACCA 3’  *C50F7.5* RT-PCR/R=  5’ TGGAGATGATGATCCAGAAG 3’ | IDT |
| *srr-6* RT-PCR/F=  5’ ATTGCCAGTGGATTCAGCAGT 3’  *srr-6* RT-PCR/R=  5’ GCCTTGAATACTTCTACGTCC 3’ | IDT |
| *Y47H9C.1* RT-PCR/F=  5’ GGACATTTCCCTACTGGAGG 3’  *Y47H9C.1* RT-PCR/R=  5’ GGTGGCCTTTGGTTTACAAAA 3’ | IDT |
| *K08C7.4* RT-PCR/F=  5’ CTCCAGGATCTGACGAAGAGG 3’  *K08C7.4* RT-PCR/R=  5’ CCCTCTGCCTCTTGCCGATG 3’ | IDT |
| *irg-5* RT-PCR/F=  5’ GATGCATCTGCGGTGAAGAAG 3’  *irg-5* RT-PCR/R=  5’ CCAGATAACCATTGTAACTCGT 3’ | IDT |
| *C33A12.19* RT-PCR/F=  5’ CTGAAAACAAGCGGAAGAAATC 3’  *C33A12.19* RT-PCR/R=  5’ CATGGAGATGCTGTATCATTG 3’ | IDT |
| *clec-52* RT-PCR/F=  5’ ATTCCTTGTTGGTTTTTCAAAG 3’  *clec-52* RT-PCR/R=  5’ ATCAGCAACTAAAGAAGTCCAC 3’ | IDT |
| *pals-39* RT-PCR/F=  5’ GTTTGCTCCGAATTCATAAAACG 3’  *pals-39* RT-PCR/R=  5’ GAGTGATGTCTTGAACGCCA 3’ | IDT |
| *mpk-2* RT-PCR/F=  5’ CGTCGGCTGAAACAATTGATAC 3’  *mpk-2* RT-PCR/R=  5’ GCCAGATAACATAGGTGGAGC 3’ | IDT |
| *C54F6.12* RT-PCR/F=  5’ GAAAAGGTTTTGACCTGCGTAAAAG  3’  *C54F6.12* RT-PCR/R=  5’ GCTCTTTTTGTCCTCAAAAGATTTG 3’ | IDT |
| *Pmyo-3*/F_=_  5’ gctagCCTGCAGGAGTGATTATAGTC  TCTGTTT 3’  *Pmyo-3*/R=  5’ taagcaGTCGACCATTTCTAGATGGA  TCTAGT 3’ | Ghazi laboratory |
| *Pgly-19*/F=  5’ gctagCCTGCAGGcgaccgccgattgattgg  gg 3’  *Pgly-19*/R=  5’ taagcaGTCGACcagaattgagagttctcaatg 3’ | Ghazi laboratory |
| *Prgef-1*/F=  5’ gctagCCTGCAGGcgcaacattgaattccgac  caagagc 3'  *Prgef-1*/R=  5’ taagcaGTCGACCATCGTCGTCGTCG  TCGATGCCGTCTTCACGA 3’ | Ghazi laboratory |
| *Pcol-12*/F=  5’ gctagCCTGCAGGtcagtatttgctattgac 3’  *Pcol-12*/R=  5’ taagcaGTCGACttttctaaaaagtaatcaaat  c 3’ | Ghazi laboratory |
| *PK08C7.4/F=*  5’ggagggtaccggtagaaaaattaaactgttcaatgctcctcaaagttttcac 3’  PK08C7.4/R=  5’ agttcttctcctttactcattcctgtaaacatgttgtttgtgaaacagttatca 3’ | IDT |
| *pPD95.75(PK08C7.4)/F=*  5’ caaacaacatgtttacaggaatgagtaaaggaga  agaacttttcactgg 3’  *pPD95.75(PK08C7.4)/R*  5’ aggagcattgaacagtttaatttttctaccggtaccct  ccaagg 3’ | IDT |
| *fmo-2(FAD)* genotyping/F*=*  5’ gccgtgaaagttctgtacatcttg 3’  *fmo-2(FAD)* genotyping/R=  5’ CGCCATCAAAGATTTCTTCCAACG 3’ | IDT |
| *fmo-2(NADPH)* genotyping/F=  5’ CACCTCAAGAAAATCTAGCAAATTT  C 3’  *fmo-2(NADPH)* genotyping/R=  5’ CCAGTTGACATCACGACCTCGTC 3’ | IDT |
